# Hantaviruses in Serbia and Montenegro

**DOI:** 10.3201/eid1206.051564

**Published:** 2006-06

**Authors:** Anna Papa, Bojana Bojovič, Antonis Antoniadis

**Affiliations:** * World Health Organization Collaborating Center for Reference and Research on Arboviruses and Haemorrhagic Fever Viruses at Aristotle University of Thessaloniki, Thessaloniki, Greece;; † Torlak Institute of Immunology and Virology, Belgrade, Serbia and Montenegro

**Keywords:** Hantavirus, Dobrava/Belgrade virus, molecular epidemiology, hemorrhagic fever with renal syndrome, dispatch

## Abstract

Hantaviruses are endemic in the Balkan Peninsula. An outbreak of hemorrhagic fever with renal syndrome occurred in 2002 in Serbia and Montenegro. The epidemiologic characteristics and genetic relatedness of Dobrava/Belgrade virus strains responsible for most cases are described.

Hantaviruses (*Bunyaviridae*) are enveloped, single-stranded, negative-sense RNA viruses with a tripartite genome consisting of a small (S), a medium (M), and a large (L) segment, which encode the nucleocapsid protein, the glycoprotein precursor and the putative RNA polymerase, respectively (*1*). Hantaviruses are transmitted to humans through aerosols of excreta from small mammals, mainly rodents, that have had silent lifelong-infections. More than 30 different hantaviruses have been distinguished so far, at least half are related to disease in humans. These viruses cause hemorrhagic fever with renal syndrome (HFRS) in Asia and Europe and hantavirus pulmonary syndrome (HPS) in America. HFRS is caused by Hantaan (HTNV), Dobrava/Belgrade (DOBV), Seoul (SEOV), and Puumala (PUUV) hantaviruses, while HPS is caused by Sin Nombre (SNV) and related viruses. Each hantavirus is associated with a specific primary rodent reservoir of the *Muridae* family; these relationships have coevolved over a long period, probably >50 million years (*1*).

HFRS is endemic in the Balkan Peninsula, where sporadic cases or outbreaks have been reported. The disease is seen during the summer and affects mainly adults (*2**,**3*), although infections in children, some fatal (*4*), have been reported. Hantaviruses associated with disease in humans in Balkans are DOBV, carried by the yellow-necked mouse (*Apodemus flavicollis*), which causes severe HFRS with a fatality rate up to 10%, and PUUV, carried by the red bank vole (*Clethrionomys glareolus*). PUUV causes nephropathia epidemica, a milder form of HFRS, with a fatality rate <1% (*3**,**5**–**8*). Recently, *A. agrarius* was found to be an additional host of DOBV, causing a milder disease than that associated with *A. flavicollis* (*9*). Additionally, Tula virus RNA was amplified from lung tissues of a European pine vole (*Pitymys subterraneus*) in Serbia (*10*).

The first probable HFRS case was reported in former Yugoslavia in 1952 (*11**,**12*); the first identified epidemic of HFRS occurred in 1961 (*13*). Some years (namely, 1961, 1967, 1979, 1986, 1989, and 1995 [2]) are characterized by increased HFRS cases. Different factors, such as weather and food abundance, could influence the dynamics of rodent populations.

The more recent large epidemic in Serbia and Montenegro occurred in 2002 with 128 laboratory-confirmed cases. The number of confirmed cases was lower in the following years. In 2003, 16 cases occurred in Serbia and 18 in Montenegro (1 fatal). In 2004, 20 cases (1 fatal) occurred in Serbia and 11 in Montenegro.

During 2002, a total of 376 serum samples from patients with suspected HFRS cases were tested in Torlak Institute, Belgrade, by indirect immunofluorescent assay (IFA) for the presence of hantavirus antibodies. IFA was performed on spot slides containing Vero E6 cells infected with HTNV, SEOV, PUUV, and DOBV. For 128 cases (77 from Serbia, 51 from Montenegro), a laboratory diagnosis of HFRS was made. Most patients (77.3%) were infected with DOBV-like viruses; the rest were infected with PUUV-like viruses. Briefly, 53 (69%) of 77 samples from Serbia and 46 (90%) of 51 from Montenegro had higher antibody titers to HTNV and DOBV than to PUUV; the other samples had higher titers to PUUV. Two Serbian patients who lived in Leskovac died. Most DOBV-like infections from Serbia occurred in the south (Leskovac, Vranje, Nis, Surdulica, Vlasina), while the PUUV-like infections occurred in the north (Vojvodina and area near the River Drina) (map of Serbia and Montenegro available from http://www.un.org/Depts/Cartographic/map/profile/yugoslav.pdf).

Thirty-one serum samples from the IFA-positive patients were sent to Aristotle University for additional testing. Samples were taken from 21 HFRS patients with a mean age of 40.3 years (21–68 years); 1 sample was obtained from a 5-month-old male infant, whose mother had HFRS at the time of delivery. Two of 21 patients died. Enzyme-linked immunosorbent assay (ELISA) to detect immunoglobulin G (IgG) and IgM antibodies to HTNV and PUUV was performed with kits by Progen (Biotechnik GmbH, Heidelberg, Germany). IgM antibodies to HTNV were detected in 18 of 21 patients; 9 also carried IgM antibodies to PUUV, although in lower titers than to HTNV (Table 1). IgG antibodies to HTNV were present in 17 of 21 patients; in 3 patients low titers of IgM antibodies to PUUV were also detected. The infant had IgG antibodies to HTNV. In 1 sample (DR) no antibodies to HTNV or PUUV were detected, although it was positive by IFA. ELISA results suggested that all 21 patients had an HTNV-like infection.

**Table 1:** ELISA and PCR results from 31 serum samples tested in this study*

Patient	Sex	Year of birth	ELISA (indexes)					PCR		
			Collection date (day of illness)	IgM HTNV	IgG HTNV	IgM PUUV	IgG PUUV	MS	MM-G1	PPT
GD	M	1979	Oct 17	2.5	1.8	Cutoff	Cutoff	Neg		
CS	M	1972	Oct 17	2.9	Neg	Cutoff	Neg			
PM	M	1958	Oct 9	Neg	1.8	Neg	1.4			
DZ	F	1951	Sep 20	3.2	2.0	Cutoff	Neg	Neg		
RD	M	1968	Aug 19	3.3	2.3	Cutoff	Cutoff	Neg		
DR	M	1958	Aug 9	Cutoff	Neg	Cutoff	Cutoff			
SS	F	1973	Aug 1	2.9	2.1	Neg	Neg			
DO	M	1956	Jul 26	1.8	2.5	1.5	1.5	Neg		Neg
RM	M	UNK	Jul 12	2.65	2.13	Cutoff	Cutoff			
VM	M	1962	Jun 14	2.7	Cutoff	1.61	Cutoff	Neg		Neg
MM	M	1936	Jun 10	2.7	2.4	Neg	Neg			
GM	M	1937	May 13	3.2	2.8	Cutoff	Cutoff	Neg		
TV	F	1964	Apr 23 (day 11)	1.6	3.3	Neg	1.7	Neg		
			Apr 24 (day 12)	1.7	3.5	Neg	2.0	DOBV	Neg	
			May 8 (day 26)	Neg	4.7	Neg	1.9			
IR	F	1981	May 17(day 8)	6.9	2.0	1.6	Cutoff	Neg		Neg
			May 24 (day 15)	6.6	2.9	1.5	Cutoff			
CJ	M	1957	May 24 (day 11)	5.0	2.5	1.5	Neg	Neg		Neg
			May 27 (day 14)	4.2	2.7	1.8	Neg			
			Oct 23 (day 5)	Neg	4.2	Neg	Neg			
TD	M	1958	Jun 11 (day 12)	4.0	1.7	1.9	2.2	Neg		Neg
			Jun 13 (day 14)	5.0	2.5	1.8	2.2			
			Jun 28 (day 29)	4.4	3.4	2.0	2.2			
GA	M	2002	Jun 19	Neg	1.8	Neg	Cutoff	Neg		Neg
SM	M	1961	Jul 3 (day 7)	3.1	1.1	Neg	Neg	Neg		Neg
			Jul 7 (day 11)	4.6	2.4	Neg	Neg			
PV	F	1972	Jun 20 (day 5)	4.3	2.0	Neg	Neg	DOBV	Neg	
MD	?	1981	Sep 18 (day 8)	4.3	1.2	Neg	Neg	DOBV	DOBV	
			Sep 30 (day 20)	4.1	5.0	Neg	Neg	Neg		

*ELISA, enzyme-linked immunosorbent assay; PCR, polymerase chain reaction; HTNV, Hantaan virus; PUUV, Puumala virus; DOBV, Dobrava/Belgrade virus; MS, primers for partial N coding regions of hantaviruses associated with rodents of the *Murinae* subfamily; MM-G1, primers for partial G1 coding regions of hantaviruses associated with rodehttp://dx.doi.org/10.3201/eidnts of the *Murinae* subfamily; PPT, primers for partial N coding regions of hantaviruses associated with rodents of the *Arvicolinae* subfamily; Unk, unknown; neg, negative; DOBV, a positive PCR band yielding Dobrava/Belgrade virus nucleotide sequences.

Viral RNA was extracted from IgM-positive samples (a sample from the neonate was also included) by using the viral RNA extraction kit (Qiagen GmbH, Hilden, Germany). Reverse transcription and nested amplification were performed with primers previously designed to detect partial S and M segment sequences from hantaviruses associated with rodents of the *Murinae* and *Arvicolinae* subfamilies (*14**,**15*). Three samples (M.D., T.V., P.V.) gave a PCR product of the expected size of 599 bp, when primers specific for the S segment of hantaviruses associated with *Murinae* rodents were used; 1 sample (MD) gave a product of 317 bp with the primers for the M segment of the same hantaviruses. No product was obtained when primers specific for the S segment of hantaviruses associated with *Arvicolinae* rodents were used. Nucleotide sequences were aligned with respective hantavirus sequences retrieved from GenBank; genetic distances were measured by the neighbor-joining method, and phylogenetic trees were constructed by using PHYLIP (Phylogeny Inference Package by J. Felsenstein [http://evolution.genetics.washington.edu/phylip.html]). The nucleotide sequences were assigned the accession numbers DQ305279-DQ305282.

Two phylogenetic trees were constructed, one for the S segment (Figure 1) and another for the M segment (Figure 2). In both trees, hantavirus strains from Serbia and Montenegro cluster with other DOBV sequences and were associated with the rodent *A. flavicollis*. In the S segment tree, sequences of this study comprise the Serbian clade in the DOBV-*A. flavicollis* cluster. In the same cluster are the Slovenian, Slovakian, and Greek clades. Sequences of this study differ by 0.3%–2.6% at the nucleotide level, with identical deduced aminoacid sequences. Genetic distances with other DOBV sequences are seen in Table 2. Concerning the M segment, a fragment of the G1-coding region of patient MD differed by 5.7% at the nucleotide level from the Slovenian DOBV strain isolated from *A. flavicollis*, with identical deduced amino acid sequences. The differences from DOBV strains from northwestern Greece were 8.5%–9.4% and 1% at nucleotide and amino acid levels, respectively.

**Figure 1:**
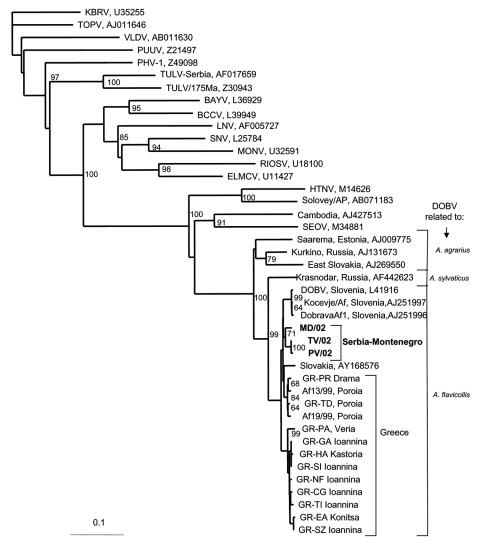
Phylogenetic tree based on partial S segment fragment showing the clustering of the sequence obtained from this study and respective representative hantavirus strains from GenBank database. The numbers indicate percentage bootstrap replicates (of 100); values <60% are not shown. Horizontal distances are proportional to the nucleotide differences. The scale bar indicates 10% nucleotide sequence divergence. Vertical distances are for clarity only. BAYV, Bayou virus; BCCV, Black Creek Canal virus; ELMCV, El Moro Canyon virus; HTNV, Hantaan virus; KBRV, Khabarovsk virus; LNV, Laguna Negra; MONV, Monongahela virus; NYV, New York virus; PHV, Prospect Hill virus; PUUV, Puumala virus; RIOSV, Rio Segundo virus; SEOV, Seoul virus; SNV, Sin Nombre virus; TOPV, Topografov virus; TULV, Tula virus; VLDV, Vladivostok virus. Accession numbers of Greek DOBV strains are AF060014–AF060024 for sequences from human cases and AJ410615 and AJ410619 from *Apodemus flavicollis* (Afl) sequences. Sequences in this study are indicated in **boldface**.

**Figure 2:**
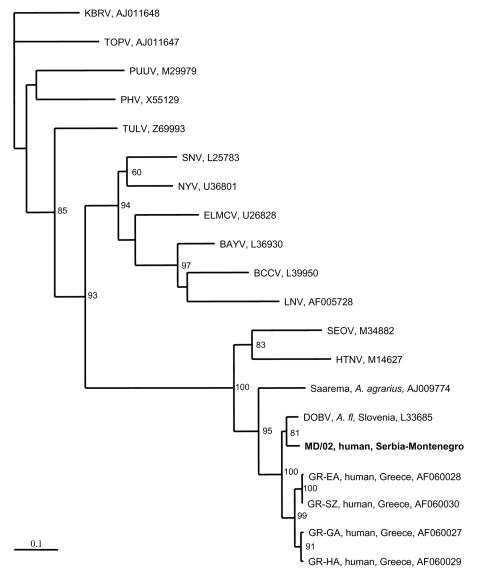
Phylogenetic tree based on partial M segment fragment showing the clustering of the sequence obtained from this study and respective representative hantavirus strains from GenBank database. The numbers indicate percentage bootstrap replicates (of 100); values <60% are not shown. Horizontal distances are proportional to the nucleotide differences. The scale bar indicates 10% nucleotide sequence divergence. Vertical distances are for clarity only. Sequences in this study are indicated in **boldface**. BAYV, Bayou virus; BCCV, Black Creek Canal virus; ELMCV, El Moro Canyon virus; HTNV, Hantaan virus; KBRV, Khabarovsk virus; LNV, Laguna Negra; MONV, Monongahela virus; NYV, New York virus; PHV, Prospect Hill virus; PUUV, Puumala virus; RIOSV, Rio Segundo virus; SEOV, Seoul virus; SNV, Sin Nombre virus; TOPV, Topografov virus; TULV, Tula virus; VLDV, Vladivostok virus.

**Table 2:** Genetic distances (%) in partial S segment fragment (364–963 nucleotides) of hantaviruses associated with *Murinae* rodents among Yugoslavian DOBV strains and representative DOBV strains related with different *Apodemus* spp. rodents*

Strain	Yugoslavian DOBV strains			*A. flavicollis* related					*A. agrarius* related		*A. sylvaticus* related
	7937/02 (PV)	5157/02 (TV)	9744/02 (MD)	GR-EA NW Greece	GR-PA NC Greece	GR-TD NE Greece	AP-Af9 NE Greece	DOBV-1 Slovenia	862 East Slovakia	Saarema Estonia	Krasnodar Russia
PV		0.3	2.6	4.9	4.9	3.0	3.2	4.0	17.5	15.6	9.5
TV			2.2	4.5	4.5	2.8	3.0	3.6	17.0	15.0	9.0
MD				4.5	4.5	2.8	3.4	3.6	16.1	15.3	9.4

*DOBV, Dobrava/Belgrade virus; –, 0.0.

Patient TV was a 38-year-old woman who lived in Vranje. Patient PV was a 29-year-old woman who lived in Leskovac. Both of these locations are in southeastern Serbia. PV died on day 6 of illness. Patient MD was living in Beograd. However, his sequences were similar to those of patients TV and PV. His travel history showed that 18 days before the onset of illness, he was on vacation in Kolasin Mountain in Montenegro, where he was probably infected. Thus, all sequences of this study were from the southern region of the country and clustered with other DOBV strains associated with *A. flavicollis* rodents. However, the involvement of other hantaviruses in the outbreak cannot be excluded.

Although the number of samples tested was limited, this study gives the first genetic information on DOBV strains circulating in Serbia and Montenegro. Further studies of both patients and small mammals in the region are needed to find out the exact epidemiology of HFRS in the country.
